# The complete mitochondrial genome of *Glyptothorax zainaensis* (Siluriformes, Sisoridae, *Glyptothorax*): genome characterization and phylogenetic analysis

**DOI:** 10.1080/23802359.2015.1137821

**Published:** 2016-02-05

**Authors:** Li Bo, Tian Zhifu, Qin Ya, Hao Meng, Zhang Jiabo

**Affiliations:** a College of Fisheries, Huazhong Agricultural University, Wuhan, China;; b Changjiang Water Resources Protection Institute, Wuhan, China

**Keywords:** Control region, *Glyptothorax zainaensis*, mitochondrial genome, phylogenetic relationship

## Abstract

*Glyptothorax zainaensis*, a small-sized benthic fish which mainly distributes in the Nujiang River, Lancangjiang river and their tributaries in China. In the present study, the complete mitochondrial genome of *G. zainaensis* was sequenced to be 16 537 bp in length, including 13 protein-coding genes, 2 ribosomal RNAs, 22 transfer RNAs, a control region and the origin of the light strand replication. The overall nucleotide composition was 31.17% A, 25.95% T, 27.48% C and 15.40% G, with an A + T bias of 57.13%. The gene composition and the structural arrangement of the *G. zainaensis* complete mitochondrial DNA were identical to most of the other vertebrates. This will provide a useful tool for understanding the genetic diversity, population structure and conservation status of *G. zainaensis* in the future.

## Introduction

Mitochondrial DNA (mtDNA) gene order was proposed to be quite conserved within vertebrates based on the gene order of the initial genome sequence (Anderson et al. 1981; Bibb et al. 1981). *Glyptothorax zainaensis* (Siluriformes: Sisoridae) is an endemic fish species which mainly distributes in the Nujiang River, Lancangjiang river and their tributaries in China. In recent years, the natural resource of this species has seriously declined, as a result of overharvesting, water contamination and especially dam construction (Jiang et al. 2007; Huang et al. 2011). In the long run, a good understanding of the genetic diversity and population structure of *G. zainaensis* is required in order to establish adequate management plans for the conservation of this species. To address these topics, we determined the complete mitochondrial genome sequence of *G. zainaensis* for the first time.

Specimens of *G. zainaensis* were collected from Yunnan Province, Nujiang River (25°51′22.81″N. 98°51′0.78″E) in March 2015 and preserved in 95% ethanol until Total genomic DNA was isolated from the caudal fin by proteinase K digestion followed by the standard phenol/chloroform method (Sambrook and Russell, 2001) and visualized on 1.5% agarose gel. Twenty sets of primers were designed for PCR amplification on the basis of aligned mitogenome sequences of *Glyptothorax trilineatus* with (Accession NC_021608.1). In order to avoid errors of assembly, the complete mtDNA genome was aligned and checked with 4 reported mtDNA genome sequences of Sisoridae species *Glyptothorax sinensis* (Accession NC_024672.1); *Glyptothorax fokiensis fokiensis* (Accession NC_018769.1); *Glyptothorax trilineatus* (Accession NC_021608.1). The assembled sequence was analyzed using the software MitoAnnotator (Iwasaki et al. 2013) and nucleotide composition was calculated by MEGA6 (Tamura et al. 2013).

The complete mtDNA sequence of *G. zainaensis* reported here has been deposited in GenBank under the accession number KU212205. The mitochondrial genome of *G. zainaensis* is a circular molecule of 16 537 nucleotides, which is similar to other vertebrates, including 13 protein-coding genes, 2 ribosomal RNA genes, 22 transfer RNA genes and a non-coding control region (D-loop). The overall nucleotide composition is 31.17%, 25.95%, 27.48% and 15.40% for A, T, C and G, with an A + T content of 57.13%, respectively. Except for a single protein-coding gene (ND6) and eight t RNA genes (t RNA*^Gln^*, t RNA*^Ala^*, t RNA*^Asn^*, t RNA*^Cys^*, t RNA*^Tyr^*, t RNA*^Ser^* (UCN), tRNA*^Glu^* and tRNA*^Pro^*) encoded on the L-strand. All the other genes were encoded on the H-strand. The first non-coding region is 888 bp between tRNA*^Pro^* and tRNA*^Phe^*, and the second one is the origin of light-strand replication, which extends up to 30 bp. It is located in a cluster of five tRNA genes (the WANCY region) between tRNA*^Asn^* and tRNA*^Cys^* gene.

Furthermore, the termination codon varies with TAA, TA, T or TAG. Virtually, all of the 13 protein-coding genes show the regular initiation codon ATG with the sole exception of COI which started with GTG. Six protein-coding genes terminated with the complete stop codon TAA (ND1, COI, ATPase8, ND4L and ND5) or TAG (ND6), while the rest ended with incomplete stop codon T (ND2, COII, COIII, ND3, ND4 and Cytb) or TA (ATPase 6)which is quite typical among mtDNA genes in other fishes (Zhou et al. 2012; Wang et al. 2013).

**Figure 1.**
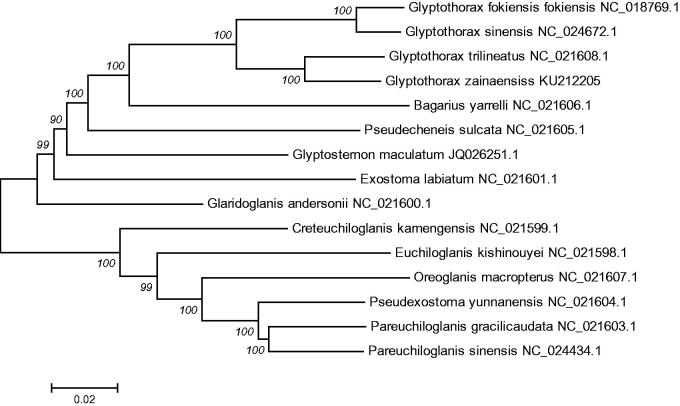
The consensus phylogenetic relationship of the *Glyptothorax zainaensis* with other Sisoridae species. *Oreoglanis macropterus*, *Pseudexostoma yunnanensis*, *Pareuchiloglanis gracilicaudata* and *Pareuchiloglanis sinensis* were used as an out-group. The numbers along the branches are Bayesian posterior probability and bootstrap values for NJ, estimated for concatenated mitochondrial protein sequences.

In addition, the mtDNA sequences of 14 species of fishes were downloaded from Gen Bank, *Oreoglanis macropterus* (Accession NC_021607.1), *Pseudexostoma yunnanensis* (Accession NC_021604.1), *Pareuchiloglanis gracilicaudata* (Accession NC_021603.1), *Pareuchiloglanis sinensis* (Accession NC_024434.1) were used as an out-group for phylogenetic analysis. Phylogenetic analyses were performed using the neighbor joining (NJ) in MEGA 6.0 (Kumar et al. 2008). The tree topologies based on complete mt DNA sequences in this study were identical and were statistically supported by high bootstrap and posterior probability values (Figure 1). The mitogenome data provided strong support that *G. zainaensis* was clustered together and form a sister group with *Glyptothorax trilineatus* (Accession NC_021608.1), *Glyptothorax sinensis* (Accession NC_024672.1), *Glyptothorax fokiensis fokiensis* (Accession NC_018769.1), *Bagarius yarrelli* (Accession NC_021606.1), *Pseudecheneis sulcata* (Accession NC_021605.1), *Glyptosternon maculatum* (Accession JQ026251.1), *Exostoma labiatum* (Accession NC_021601.1), *Glaridoglanis andersonii* (Accession NC_021600.1). The phylogenetic analyses yielded convincing evidence that the *G. zainaensis* is located at the bottom position in *Glyptothorax* species, and that the tandem repeats might be a feature of the ancestral teleost lineage.
